# Oral health and the presence of infectious microorganisms in hospitalized patients: a preliminary observational study

**DOI:** 10.1080/07853890.2022.2092895

**Published:** 2022-09-08

**Authors:** Adriana Silva da Costa Cruz, Yara Peixoto Fidelis, Danielly de Mendonça Guimarães, Herick Sampaio Muller, Vicente de Paulo Martins, Erica Negrini Lia

**Affiliations:** a Postgraduate Program in Dentistry, University of Brasília, Brasília, Brazil; b Faculty of Health Sciences, Dentistry Department, University of Brasília, Brasília, Brazil; c Laboratory of Molecular Analysis of Pathogens, Institute of Biological Sciences, University of Brasília, Brasília, Brazil

**Keywords:** Hospitalization, mouth, oral health, microbiota

## Abstract

**Objective:**

Characterise oral health, and the presence in the oral cavity of pathogenic non-oral microorganisms potentially associated with nosocomial infections and antimicrobial resistance in non-intubated patients admitted to a Brazilian university hospital.

**Materials and methods:**

An intraoral examination and oral swab were performed on hospitalized individuals at three different times, T1 (within 48 h of hospitalization), T2 (48 h after T1) and T3 (7 days after hospitalization). The oral health status was defined by the Oral Health Assessment Tool (OHAT) and Tongue Coating Status (TCS). The swabs were processed and microorganisms potentially related to nosocomial infections were phenotypically identified through colony morphology, staining and microscopy.

**Results:**

The most prevalent microorganisms were *Escherichia coli*, *Enterococcus* spp., *Enterobacter* spp., *Pseudomonas* spp., *Candida albicans* and *Staphylococcus aureus*. The oral health status was considered median, and the tongue coating index was considered high throughout the study period. The prevalence of potentially pathogenic non-oral microorganisms was high and constant from the first 48 h to the seventh day of hospitalization.

**Conclusions:**

The results point out that the mouth can act as a reservoir of epidemiologically important pathogens within hospital settings, even in patients without mechanical ventilation, thus increasing the risk of nosocomial infections in susceptible individuals.

**KEY MESSAGES**
- The present study investigated the oral health status and the presence of pathogenic non-oral microorganisms in the oral cavity of patients hospitalized in the ward, non-intubated and mostly independent of self-care.
- The presence in the mouth of microorganisms related to the epidemiology of nosocomial infections and resistance to antimicrobials was high and constant from the first 48 h to the 7th day of hospitalization.
- The results of this study point out that the mouth can act as a reservoir of epidemiologically important pathogens within hospital settings even in patients without mechanical ventilation, increasing the risk of nosocomial infections in susceptible individuals.

## 1. Introduction

The human mouth is naturally colonized by a diversified microbiota, composed of about 700 species of bacteria, in addition to fungi, archaea, viruses and protozoa [1], which exhibits commensalism, symbiosis and pathogenic relations with the host [2]. Several factors alter both the homeostasis and the composition of the oral microbiome, such as chemical interactions with enzymes or microorganisms, decreased salivary flow, reduced production of immunoglobulins, and presence of proteases and neuraminidase associated with gingivitis and periodontal disease [3]. These conditions are associated with the occurrence of early colonization of the oral cavity by Gram-negative microorganisms and strains resistant to multiple antimicrobials, which can occur during a period of hospitalization [3].

The study conducted by Cecon et al. in 2010 showed time-dependent colonization of the mouth by *Enterobacteriaceae*, *Staphylococcus aureus* and *Candida* spp. in comatose patients who did not receive oral hygiene [4]. When self-care with oral hygiene is compromised, such as in hospitalizations in which the patient is restricted to bed or is unconscious, oral health deterioration occurs due to the accumulation of oral biofilm, deposited on the teeth and dental prostheses [5,6].

Considering that the mouth has a direct relationship with both respiratory and digestive tracts and that it gathers all the ideal conditions of temperature, humidity and nutrition, there may be microorganisms’ translocation between such means, especially in individuals with poor oral hygiene, intubated and systemically weakened [2]. Studies have shown that the mouth acts as a reservoir of mandatory anaerobic respiratory pathogens (belonging to the *Prevotella* and *Fusobacterium* genera) in institutionalized elderly and hospitalized individuals who have poor oral hygiene, which seems to be a risk factor for the development of diseases, such as aspiration pneumonia [7–9]. Colonisation of endotracheal tubes by pathogenic microorganisms, related to pneumonia from the mouth, has been reported in patients on mechanical ventilation [10].

A study demonstrated that the saliva and oral biofilm of non-intubated patients were highly colonized by respiratory pathogens in approximately 14 days of hospitalization that preceded elective myocardial revascularization surgery [11]. These data show that the mouth can act as a reservoir of mandatory anaerobic respiratory pathogens even in patients without mechanical ventilation, increasing the risk of nosocomial pneumonia in susceptible individuals [11].

Considering the scarcity of studies on the pathogenic non-oral microorganisms in non-intubated hospitalized individuals, the objective of our study was to evaluate the oral health status and the presence of aerobic microorganisms potentially associated with hospital infections and resistance to antimicrobials in the oral cavity of individuals admitted to a Brazilian university hospital.

## 2. Methodology

### 2.1. Study design

This is an observational, longitudinal study with before-after analysis, developed at the University Hospital of Brasília (Brazil). The study was conducted in accordance with the Declaration of Helsinki and was approved by the Human Research Ethics Committee of the Medicine School of the University of Brasília (Certificate of Presentations of Ethical Appreciation number 87378818.7.0000.5558, technical opinion number 2.628.620).

### 2.2. Study population

The study was carried out between the months of July and December 2018, with patients hospitalized in the Medical Clinic Ward of the University Hospital of Brasília, Brasília, Brazil. Participants received clarifications about the research and signed the free and informed consent form.

Participants were included if aged 18 years or over, hospitalized for less than 48 h and without cognitive impairment. They were excluded due to immunosuppression (under chemotherapy, post-transplant, HIV) and if they had a history of recent hospitalizations that occurred in the last 30 days.

### 2.3. Initial interview and clinical record data

Participants were interviewed about their oral hygiene routine performed at the hospital, food route used during hospitalization, alcoholism, smoking and complaints related to oral health.

By consulting the medical record, data were collected on the reasons for hospitalization, presence of comorbidities and medication prescribed during hospitalization and continuous use.

### 2.4. Oral health assessment

Oral health assessment was performed by a single calibrated dental surgeon, in a hospital bed, under artificial lighting and in accordance with all biosafety standards. The intraoral examination was performed in three moments, the first happened within 48 h after hospital admission (T1); the second, was performed 48 h after T1; and the third, was performed 7 days after hospital admission (T3).

The OHAT (Oral Health Assessment Tool) and the TCS (Tongue Coating Status) were used to determine the oral health status. The intra-examiner Kappa index obtained for OHAT was 0.83 and for TCI was 0.74.

The OHAT assesses soft tissues, saliva quality, presence and appearance of natural teeth and prostheses, oral hygiene pattern and presence of pain [12]. Each item is scored from 0 to 2, with the lowest score representing no change and gradually increasing the score according to the presence of changes. The sum of the scores obtained in each of the items defines the final score, which varies between 0 (very healthy) and 16 (very sick) [12].

The TCS classifies the tongue coating according to its length [13]. A coated tongue can be associated with a range of conditions and occurs when there is an accumulation of a layer composed by bacteria, food matter and dead cells on the tongue surface. For this purpose, the division of the tongue body into three-thirds is considered and the presence or absence of tongue coating is evaluated, without considering its thickness. Scores vary from 0 to 3, with 0 indicating no visible coating, 1 indicating less than ⅓ of the tongue body, 2 indicating less than ⅔ and 3 greater than ⅔ of the tongue body covered by the coating [13].

### 2.5. Collection of microbiological samples and identification of the pathogenic non-oral microorganisms

In order to identify the presence, in the oral cavity, of non-oral microorganisms potentially related to the occurrence of antimicrobial resistance and hospital infections, microbiological samples were collected by rubbing a sterile swab on the unilateral vestibular surface of lower molars when present, unilateral vestibular mucosa in the region of lower molars and tongue body. Collections were always performed in the morning, approximately one hour after feeding. The swabs, contained in individual closed packages without a culture medium, were transported in a refrigerated container to the Laboratory of Molecular Analysis of Pathogens (LAMP) of the Institute of Biological Sciences at the University of Brasília, where they were processed on the same day of collection.

The samples contained in the swabs were individually homogenized in 1 mL of sterile saline and centrifuged at 10,000 × *g* for 1 min and 30 s at room temperature. The 800-µL volume of the supernatant was discarded, and the precipitate, contained in the remaining volume, was homogenized again by pipetting in order to perform seeding by exhaustion in plates with chromogenic culture media CHROmagar™ *Candida* (BD, Germany) and CHROmagar™ Orientation (BD, Germany).

CHROmagar™ Orientation is used for isolation and differentiation of urinary tract pathogens, but can also be used to differentiate various microorganisms in other infected areas [14]. Its composition has chromogenic substrates that reveal the metabolic enzymes of the microorganisms. It allows full differentiation of the pathogens by different colours and the typical appearance of each microorganism on the plate [14]. CHROmagar™ *Candida* is used for isolation and differentiation of major clinical-significant *Candida* species. It provides intense colony colouration helping to differentiate species with high specificity and sensitivity for major *Candida* species [15].

The microorganisms of the study were identified by means of colony morphology, staining and microscopy. The microorganisms found were isolated and a new inoculation on the same chromogenic culture media was performed to confirm their identification.

### 2.6. Statistical analysis

The data were presented in the form of descriptive statistics such as mean, standard deviation, absolute distribution and percentage of the variables studied. In order to evaluate the effect of the length of hospitalization on oral health indexes (OHAT and TCS) the Friedman test was used, followed by the Dunn post-test, and, for that purpose, the statistical software GraphPad Prism 5.0a Software was used (GraphPad Software Inc., San Diego, CA, USA).

In order to assess differences in the proportion of microorganisms found in each collection time, the Cochran’s *Q* test was used for related groups and to perform an analysis of the association between the presence of microorganisms and the oral health condition, the Pearson’s Chi-square test was performed with continuity correction when necessary. Both analyzes were performed using the statistical software IBM SPSS (Statistical Package for the Social Sciences) 23, 2015.

## 3. Results

**Figure 1.**
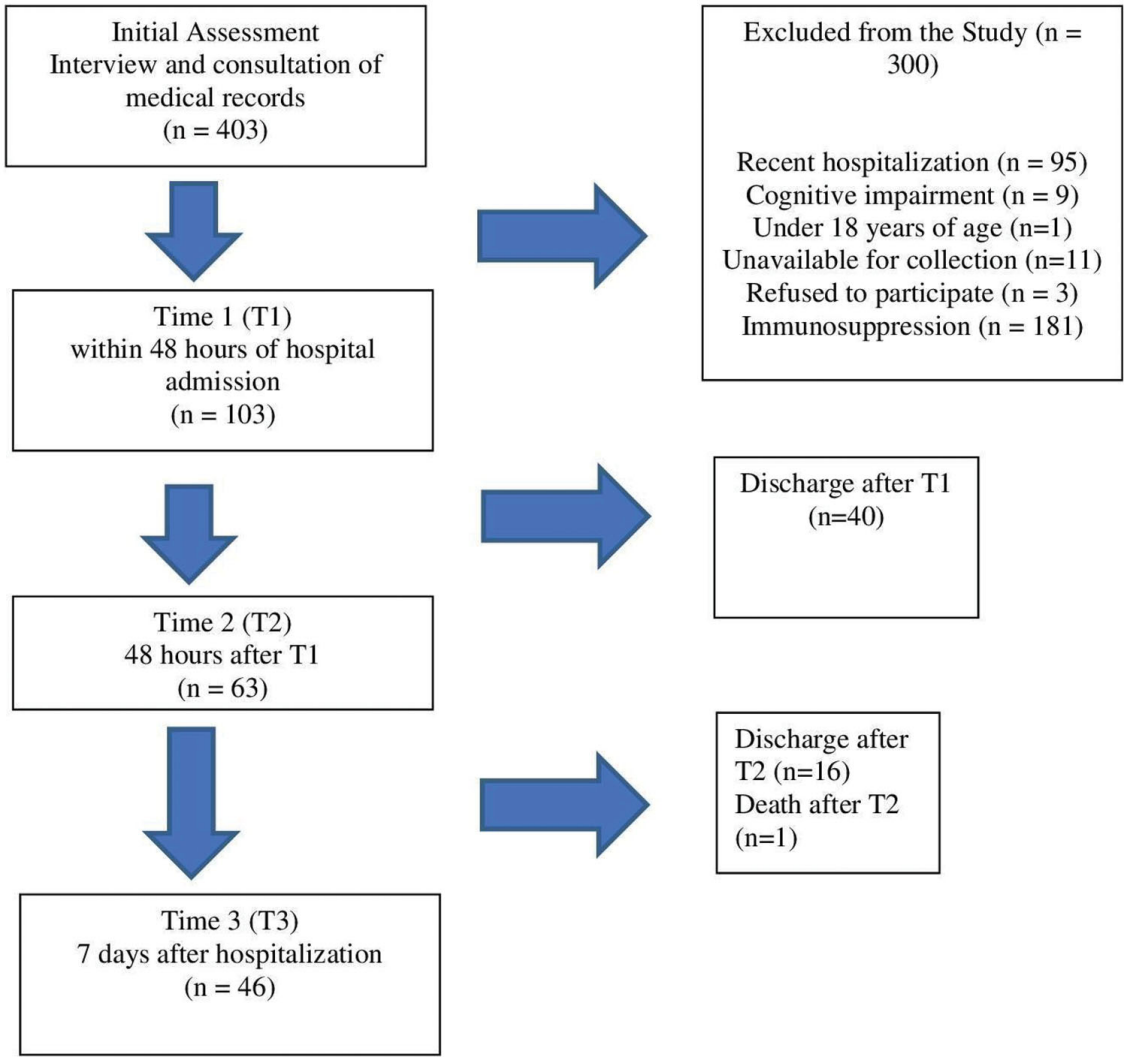
Flowchart of the clinical phase of the study.

Initially, 403 patients and their medical records were evaluated and 300 were excluded. Out of the 103 patients assessed at T1, 63 remained hospitalized at T2 and 46 at T3. (Figure 1). Table 1 describes the characterization of the population assessed in the study at T1. At T1, the majority of the patients were women (62.1%), no smokers (88%), were under physiological oral feeding (98.1%), and did not need assistance for oral hygiene (95.1%). About 40% of the patients performed oral hygiene three times per day. The average number of teeth was 13 (±12), and one-third of the patients were edentulous. About 48.5% of the patients were users of a removable dental prosthesis, and 50% of them did not remove it during sleep. Anti-hypertensive and anticoagulant drugs, besides diuretics, and antimicrobials were the main drugs utilized during hospitalization. Xerostomia was the principal oral complaint. The prevalence of oral lesions was 25.2% at T1 (ulcers, leukoplakia, candidiasis, fibroma, and hyperplasia); 26.9% in T2 and 19.5% in T3. Five patients, who did not present oral lesions at the first examination (T1), developed oral ulcers throughout the 7-day follow-up.

**Table 1.** Characterization of the sample composed of participants hospitalized in the Medical Clinic Unit of University Hospital of Brasília from July to December 2018, in T1.

	*n*	%	Mean (SD)
Sex			
Male	39	37.9	
Female	64	62.1	
Age (years)			53 (±17)
Smoking			
Yes	12	11.6	
No	91	88.3	
Route of feeding			
Physiological oral route	101	98.1	
Other	2	1.9	
Number of teeth present			13 (±12)
Total edentulism	34	33	
Removable prosthesis user			
Yes	50	48.5	
No	53	51.5	
Remove the prosthesis to sleep (*n* = 50)			
Yes	9	18	
No	41	82	
Need for oral hygiene assistance			
Yes	5	4.8	
No	98	95.1	
Daily frequency of oral hygiene			
No	8	7.8	
1×	15	14.6	
2×	39	37.9	
3× or more	41	39.8	
Main medications used			
Anti-hypertensive	58	56.3	
Anticoagulants	51	49.5	
Diuretics	40	38.8	
Antibiotics	33	32.0	
Hypoglycemic	26	25.2	
Corticosteroids	20	19.4	
Statins	20	19.4	
Main oral health complaints			
Xerostomia	55	53.4	
Chewing difficulty	28	27.2	
Halitosis	22	21.4	
Decayed or fractured tooth	18	17.5	
Decreased taste	17	16.5	
Gingival bleeding	15	14.6	
Most common oral lesions			
Ulcers	6	5.8	
Leukoplakia	5	4.8	
Candidosis	3	2.9	
Fibroma	3	2.9	
Hyperplasia	3	2.9	

Mean and standard deviation (SD) or absolute distribution (*n*) and percentage (%) of responses (*n* = 103).

Table 2 shows the OHAT and TCS indexes of patients who remained hospitalized in the three assessment periods. The median of the OHAT index was 6.0, and the mean and standard deviation was 6.2 at T1. The OHAT index improved throughout hospitalization. The TCS index was high and remained constant during the evaluation period, as well as oral hygiene.

**Table 2.** Oral Health Assessment Tool (OHAT), Tongue Coating Status (TCS) and Oral Hygiene (isolated from OHAT) of the participants hospitalized at the Medical Clinic Unit of University Hospital of Brasília in the three times of the study, from July to December 2018.

	T1 (*n* = 46)	T2 (*n* = 46)	T3 (*n* = 46)	*p*-Value
OHAT	6.2	5.9	5.8	.036*
	(±1.8)	(±2.1)	(±2.3)	
TCS	2.4	2.3	2.5	.441
	(±0.9)	(±0.8)	(±0.7)	
ORAL HYGIENE	1.3	1.3	1.2	.417
	(±0.8)	(±0.6)	(±0.6)	

Data presented as mean and standard deviation. Friedman’s Analysis of Variance; *p < .05.

In T1, 103 biological samples were collected, in T2, 63 samples and in T3, 46 samples, totalling 212 samples. Each sample was inoculated on two plates with culture media CHROmagar™ Candida and CHROmagar™ Orientation. A total of 882 microbial colonies were identified in the 212 samples collected.

Table 3 shows the sample frequencies with positive results for each microorganism identified at T1, T2 and T3. The number of microorganisms identified over the three hospitalization times remained stable, with no significant differences. The most prevalent microorganisms were *E. coli*, *Enterococcus* spp., *Enterobacter* spp., *Pseudomonas* spp., *Candida albicans* and *S. aureus*.

**Table 3.** Absolute and percentage distribution of microorganisms present in patients admitted to the Medical Clinic Ward at University Hospital of Brasilia in the three collection times (2018).

	T1	T2	T3	p*
	*n* (%)	*n* (%)	*n* (%)	
*E. coli*	37 (80.4)	36 (78.6)	34 (73.9)	.584
*Enterococcus* spp.	32 (69.6)	32 (69.6)	34 (73.9)	.641
*Enterobacter* spp.	28 (60.9)	24 (52.2)	21 (45.7)	.214
*Pseudomonas* spp.	24 (52.2)	25 (54.3)	25 (54.3)	.951
*C. albicans*	24 (52.2)	23 (50.0)	21 (45.7)	.627
*S. aureus*	18 (39.1)	19 (41.3)	15 (32.6)	.444
*Candida tropicalis*	11 (23.9)	7 (15.2)	9 (19.6)	.368
*Klebsiella* spp.	8 (17.4)	6 (13.0)	3 (6.5)	.232
*C. glabrata*	5 (10.9)	9 (17.4)	9 (17.4)	.472
*C. krusei*	5 (10.9)	7 (15.2)	5 (10.9)	.695
*Proteus* spp.	1 (2.2)	1 (2.2)	3 (6.5)	.135
*Streptococcus* spp.	0 (0.0)	1 (2.2)	2 (4.3)	.223
Total	46	46	46	

*Cochran’s *Q* test.

Table 5 shows the frequency of microorganisms according to the oral health status, using the median of OHAT value. At T1, patients with poorer oral health (OHAT > 6) had a higher frequency of *S. aureus*, *Candida krusei* and *Candida glabrata*, while patients with OHAT ≤6 were 5.4 times more likely to have *Escherichia coli* in relation to patients with higher OHAT values (Table 4). At T2, *S. aureus* and *C*. *krusei* were more frequent in patients with OHAT >6, while *Enterococcus* spp. was more frequent in patients with OHAT ≤6 (Table 5). In T3, there was no statistically significant difference in the frequency of the microorganisms found, according to the division of the OHAT index.

**Table 4.** Analysis of cross-sectional association of microorganisms in relation to the OHAT index less than or equal to 6 and greater than 6 of patients admitted to T1 at the Medical Clinic Ward of the University Hospital of Brasília (HUB) (2018).

	OHAT				
	≤6	>6			
	*n* (%)	*n* (%)	p*	RC	IC (95%)
*E. coli*	61 (93.8)	28 (73.7)	.004	5.446	1.572–18.874
*Enterobacter* spp.	34 (52.3)	17 (44.7)	.458	1.355	0.607–3.026
*S. aureus*	14 (21.5)	15 (39.5)	.049	0.421	0.175–0.998
*Streptococcus* spp	1 (1.5)	0 (0.0)	1.000		
*S. saprophyticus*	0 (0.0)	0 (0.0)			
*Enterococcus* spp.	55 (84.6)	27 (71.1)	.099	2.241	0.847–5.925
*Pseudomonas* ssp.	35 (53.8)	14 (36.8)	.095	2.000	0.881–4.541
*Proteus* spp.	3 (4.6)	4 (10.5)	.457	0.411	0.087–1.946
*Klebsiella* spp.	10 (15.4)	3 (7.9)	.425	2.121	0.546–8.248
*Citrobacter* spp.	0 (0.0)	0 (0.0)			
*C. albicans*	23 (35.4)	19 (50.0)	.145	0.548	0.243–1.236
*C. tropicalis*	10 (15.4)	10 (26.3)	.176	0.509	0.190–1.367
*C. krusei*	1 (1.5)	4 (10.5)	.041	0.133	0.014–0.926
*C. glabrata*	5 (7.7)	8 (21.1)	.049	0.313	0.094–0.979
Total	65 (100.0)	38 (100.0)			

*Pearson’s Chi-squared test.

**Table 5.** Analysis of the cross-sectional association of microorganisms in relation to the OHAT index less than or equal to 6 and greater than 6 of patients admitted to T2 at the Medical Clinic Ward of the University Hospital of Brasília (2018).

	OHAT		p*	RC	IC (95%)
	≤6	>6			
	*n* (%)	*n* (%)			
*E. coli*	35 (87.5)	16 (69.6)	.158	3.063	0.842–11.138
*Enterobacter* spp.	19 (47.5)	12 (52.2)	.721	0.829	0.297–2.316
*S. aureus*	12 (30.0)	13 (56.5)	.038	0.330	0.114–0.958
*Streptococcus* spp	1 (2.5)	0 (0.0)	1.000		
*S. saprophyticus*	0 (0.0)	0 (0.0)			
*Enterococcus* spp.	34 (85.0)	14 (60.9)	.030	3.643	1.091–12.168
*Pseudomonas* spp.	22 (55.0)	10 (43.5)	.378	1.589	0.565–4.465
*Proteus* spp.	2 (5.0)	2 (8.7)	.966	0.553	0.072–4.213
*Klebsiella* spp.	3 (7.5)	3 (13.0)	.783	0.541	0.100–2.930
*Citrobacter* spp.	0 (0.0)	0 (0.0)			
*C. albicans*	18 (45.0)	10 (43.5)	.907	1.064	0.378–2.989
*C. tropicalis*	8 (20.0)	3 (13.0)	.722	1.667	0.395–7.033
*C. krusei*	2 (5.0)	5 (21.7)	.042	0.189	0.033–0.972
*C. glabrata*	5 (12.5)	7 (30.4)	.158	0.327	0.090–1.188
Total	40 (100.0)	23 (100.0)			

*Pearson’s Chi-squared test.

## 4. Discussion

The present study investigated the oral health status and the presence of pathogenic non-oral microorganisms in the oral cavity of patients hospitalized in the ward, non-intubated and mostly independent of self-care. The oral health status presented mean values that remained from the second to the seventh day of hospitalization. The presence of microorganisms related to the epidemiology of nosocomial infections and resistance to antimicrobials was high and constant during the three times of the study.

In general, the most prevalent microorganisms in our study were *E. coli*, *Enterococcus* spp., *Enterobacter* spp., *Pseudomonas* spp., *Candida albicans* and *S. aureus*. They are all microorganisms that have strains resistant to antimicrobials associated with difficult-to-control nosocomial infections and are, therefore, important from an epidemiological point of view in the worrying scenario of the growing prevalence of antimicrobial resistance worldwide [16]. Corroborating these results, findings similar to ours were presented in a study conducted in a hospital in Curitiba (Brazil), with samples collected from the tongue body of patients hospitalized in the intensive care unit (ICU), in which a variety of gram-negative microorganisms, related to nosocomial pneumonia and other hospital infections as well, were identified in three collection times (within 24 h, 72 h and 120 h of admission to the ICU) [17].

Some microorganisms identified in our study, such as *E. coli* and *S. aureus*, had strains with broad antimicrobial resistance described in the latest WHO report published in 2020. This report presented data collected from 78 countries between May and July 2019 and high rates of resistance of various microorganisms to antimicrobials used to treat common bacterial infections were observed. For *E. coli*, the rate of resistance to ciprofloxacin, an antibiotic commonly used to treat urinary infections, ranged from 8.4% to 92.9% in at least 33 countries [16]. The average rate of infections by methicillin-resistant *S. aureus* (MRSA) was 12.11% and by *E. coli* resistant to the 3rd generation of cephalosporins was 36% [16].

*Escherichia coli*, *Enterobacter* spp., *Pseudomonas* spp. and *S. aureus* are pathogens known for their involvement in respiratory tract infections acquired within hospital settings [18]. *Pseudomonas* spp. are Gram-negative bacilli that rarely infect healthy patients [19], however, in addition to respiratory infections, they can also cause urinary infections or opportunistic bacteraemia acquired mainly by burn victims or those under mechanical ventilation [20].

*Candida albicans* are commensal organisms that inhabit the oral cavity, gastrointestinal tract and, sometimes, the skin. Under normal conditions, they are non-pathogenic microorganisms, but in immunocompromised patients, they are responsible for a large part of systemic fungal infections that are associated with a high mortality rate [21,22].

Some of these microorganisms, identified in our study, are part of a group of bacteria collectively identified by the acronym ESKAPE [23]. This acronym represents six pathogens that have shown increasing virulence and are related to nosocomial infections and resistance to multiple antibacterials. They are *Enterococcus faecium*, *S. aureus*, *Klebsiella pneumoniae*, *Acinetobacter baumannii*, *Pseudomonas aeruginosa* and *Enterobacter* spp [23,24]. This group of pathogens is responsible for most nosocomial infections and has been listed by the World Health Organisation as a priority in research for the development of new antibiotics [25].

The microbial profile found in our study may come from community colonization prior to contact with the hospital environment and early colonization from the hospital environment in less than 48 h of hospitalization. Microorganisms were identified in the first moment of our study, and their presence remained constant until the seventh day of hospitalization. In Tuon et al.’s study, the occurrence of MRSA and carbapenem-resistant *Enterobacteria* were also recorded in the initial sample, considering that colonization may have occurred on the patient’s admission to emergency care or during recent medical care [3].

There are studies demonstrating that Staphylococcus spp. can be isolated in the oral cavity of healthy individuals [26]. In immunocompetent adult individuals with the gingival-periodontal disease, *Staphylococcus* spp. and *Candida* spp. were isolated in high numbers and the prevalence was even higher in patients who used dental appliances [27]. These studies suggest that these microorganisms can colonize the oral cavity regularly, but it is not well established if it is a transient presence [26,27].

In our study, the microbiological results found may indicate the existence of a transient microbiota that may reach a greater or lesser degree of permanence in the mouth, depending on the conditions of the host. Ewan et al.’s study indicated that, in frail patients, hospital pathogens may be present at the time of hospital admission [18], which may have happened to the participants in our study. In addition, we found a higher frequency of *S. aureus* and some *Candida* species in patients whose oral health was considered worse, possibly showing a tendency of associations between these microorganisms. There are studies showing the interaction between *Candida* species and bacteria, such as *S. aureus* and *P. aeruginosa*, with the formation of polymicrobial biofilms in which the relationship between microorganisms occurs synergistically, contributing to greater virulence, mutations and greater resistance to antimicrobials from the microorganisms involved [28–30].

Although potentially pathogenic microorganisms were detected in our study in the first 48 h after hospital admission, there were no significant changes in their prevalence at the three observation times. A study with non-intubated elderly hospitalized for 14 days concluded that factors related to the host, such as age, degree of fragility and comorbidities, had a greater impact on the composition of the oropharynx microbiota than the length of hospitalization [18]. The same study also showed that there was relative stability of this microbiota during hospitalization period [18]. Another study compared a group of unconscious patients and another group with preserved ability to perform their oral hygiene and found a progressive increase in the count of microorganisms in the first group and not in the second over up to seven days [4]. These factors may explain the absence of important variations in the microbial load during the three times of our study, for the sample was mostly composed of independent participants to perform self-care.

The microbiological findings of our study support the idea that, in non-intubated patients, the microorganisms present in the oral cavity can represent a risk for nosocomial infections. A study carried out with non-intubated patients, hospitalized due to lower limb fractures, demonstrated the relationship between nosocomial pneumonia and oral microbiota [31]. The occurrence of pneumonia was associated with the presence of two or more positive oral samples for *S. aureus*, MRSA, *E. coli* or *P. aeruginosa*, at any time of hospitalization, but it was not associated with oral health in certain aspects such as the number of teeth or presence of a large amount of dental biofilm in teeth and dentures [31].

In relation to oral health, the most reported complaint by the study participants was xerostomia, followed by chewing difficulty and halitosis. The use of a large number of medications during hospitalization may be related to reports of xerostomia and halitosis [32]. Although we did not measure the salivary flow, this complaint may point out its reduction. There are studies showing the relationship between hyposalivation and increased oral colonization by microorganisms, such as *Candida* spp [33,34]. In our study, a prevalence of *C. albicans* was found in approximately 50% of the samples at the three collection times, which may indicate some relationship with the xerostomia reported by the patients. The chewing difficulty presented is probably related to a large number of totally edentulous participants, of whom, a quarter did not have a dental prosthesis. In our study, neither the worsening of oral health detectable by the indexes used was identified, nor the worsening of oral hygiene itself during the three assessment times. However, findings in the literature reveal worsening of oral hygiene with increased accumulation of bacterial biofilm and tongue coating during a 72-h to a 14-day hospitalization period [6,35–38]. Available studies in the literature deal with patients hospitalized in ICUs, therefore, one of the factors suggested to explain our results was the autonomy presented by most patients to perform their own oral hygiene, for only five of them (4.85%) needed assistance to perform this task. In a study conducted with patients hospitalized for fractures of the lower and non-ventilated limbs, there was a greater increase in the dental biofilm score among the most dependent, the ones with decreased mobility, the most fragile and the ones with dementia [36].

In the present study, about 25% of the participants presented oral lesions in each of the three moments of the evaluation. A study carried out with patients hospitalized for infectious diseases reported a prevalence of oral lesions in 84.3% of them [39]. Although our study showed a lower prevalence of oral lesions than the one mentioned above, which probably occurred due to the different profile of the sample, it can still be considered a high prevalence, which demonstrates the need for a dental surgeon in a multidisciplinary team within hospital settings, as most of these injuries cause considerable discomfort, reduced quality of life for the patient and the need of specific attention.

The oral health status remained median between the second and the seventh day of hospitalization. The tongue coating index was considered high throughout the follow-up period. The prevalence of potentially pathogenic microorganisms and related to the transmission of resistance to antimicrobials in the mouth was high from the first 48 h to the seventh day of hospitalization. Although the composition of these microorganisms has not undergone significant changes during hospitalization, the identification of microorganisms related to nosocomial infections and resistance to antimicrobials, may suggest the occurrence of previous or early colonization of the oral cavity.

Among the limitations of our study, we can mention the loss of follow-up of participants due to hospital discharge or death. In addition, the index used to measure patients’ oral health conditions evaluated categories that did not change for seven days, such as the number of teeth and the use of removable prostheses. These factors may have affected the results of the study as we could not detect the worsening in oral health and hygiene during the three assessment times. Furthermore, although the chosen culture media are considered to have good sensitivity and specificity for isolation and identification of most microorganisms at the species or genus level, there is a group of four Enterobacteriaceae (*Klebsiella* spp., *Citrobacter* spp., *Enterobacter* spp. and *Serratia* spp.) that cannot be differentiated among them [14,40,41], what may affect the accuracy of part of the results. However, considering these four microorganisms as a group, the accuracy in identifying pathogenic anaerobic microorganisms by the chromogenic culture medium is between 96% and 100% compared to other methods of phenotypic identification (colony morphology and biochemical tests) and molecular methods such as PCR, genetic and protein sequencing [14,15,40–42].

The results of this study point out that the mouth can act as a reservoir of epidemiologically important pathogens within hospital settings even in patients without mechanical ventilation, increasing the risk of nosocomial infections in susceptible individuals.

The present study is the first part of a larger research that intends to perform the isolation and differentiation of all the found microorganisms using MALDI-TOF mass spectrometry or molecular methods such as polymerase chain reaction (PCR) and genetic sequencing. The aim is to evaluate the susceptibility of each species to chlorhexidine. The minimal inhibitory concentration for chlorhexidine will be determined for all the depicted isolates. Species with altered susceptibility to chlorhexidine will be submitted to antibiogram analysis to identify possible resistance to antimicrobials for clinical use in humans.

## 5. Conclusion

The oral health status was considered average, and the tongue coating index was considered high throughout the study period. The prevalence of potentially pathogenic non-oral microorganisms was high and constant from the first 48 h to the seventh day of hospitalization. It may suggest colonization of the mouth before hospitalization or during the first hours within hospital settings.

## Data Availability

The datasets used and/or analyzed during the current study are available from the corresponding author on reasonable request and were deposited at the Open Science Framework (OSF) (https://osf.io/dp7qb/?view_only=aed524399f494421b12eb71876ce6934).
